# G Protein-Coupled Estrogen Receptor 1 Knockout Deteriorates MK-801-Induced Learning and Memory Impairment in Mice

**DOI:** 10.3389/fnbeh.2020.00157

**Published:** 2020-11-26

**Authors:** Chun Zhang, Qiang Liu, Chun-Yang Yu, Feng Wang, Yu Shao, Kui-Sheng Sun, Tao Sun, Juan Liu

**Affiliations:** ^1^Ningxia Key Laboratory of Cerebrocranial Diseases, Ningxia Medical University, Yinchuan, China; ^2^Institute of Basic Medical Sciences, School of Basic Medical Sciences, Ningxia Medical University, Yinchuan, China

**Keywords:** schizophrenia, MK-801, behavioral change, intellicage, learning and memory capacity

## Abstract

The role of estrogen receptors in neuroprotection and cognition has been extensively studied in humans over the past 20 years. Recently, studies have shifted their focus to the use of selective estrogen receptor modulators in the treatment of mental illnesses in the central nervous system. We conducted this study to test the behavioral changes shown by G protein-coupled estrogen receptor 1 knockout (GPER1 KO) and wild-type (WT) mice with MK-801-induced schizophrenia (SZ). GPER1 KO and WT mice received intraperitoneal injections of MK-801 for 14 continuous days. Behavioral, learning and memory, and social interaction changes were evaluated by using the IntelliCage system, open-field, three-chamber social interaction, and novel object recognition tests (NORT). The protein expression levels of the NR2B/CaMKII/CREB signaling pathway were tested *via* Western blot analysis. The KO SZ group was more likely to show impaired long-term learning and memory function than the WT SZ group. Learning and memory functions were also impaired in the KO Con group. MK-801 administration to the GPER1-KO and WT groups resulted in memory deficiencies and declining learning capabilities. GPER1 deficiency downregulated the expression levels of proteins related to the NR2B/CaMKII/CREB signaling pathway. Our study suggested that GPER1 played an important role in cognitive, learning, and memory functions in the MK-801-induced mouse model of SZ. The mechanism of this role might partially involve the downregulation of the proteins related to the NR2B/CaMKII/CREB signaling pathway. Further studies should focus on the effect of GPER1 on the pathogenesis of SZ *in vivo* and *in vitro*.

## Introduction

Schizophrenia (SZ) is a neurological disorder that affects approximately 0.7–1% of the population (Valton et al., 2017; Marder and Cannon, 2019). The symptoms of this disorder include delusions, movement disorders, anhedonia, hallucinations, and cognitive deficits (Marder and Cannon, 2019). SZ is a chronic disease that negatively affects all aspects of a patient’s life. Although most patients with SZ can live in a community, the outcome of this mental disease is generally poor, and some patients with SZ may require chronic institutional care (Borelli and Solari, 2019).

The role of estrogens and estrogen receptors in neuroprotection and cognition has been extensively studied in humans over the past 20 years. Results obtained with rodent models suggest that estrogen treatment can improve memory and cognition functions in various neurological disorders (Bean et al., 2014). For example, a previous study showed that postmenopausal women receiving estrogen therapy may experience improved cognition functions, including verbal memory, oral expression, and information processing (Persad et al., 2009). These findings suggest that estrogens can be used in the clinical therapy of patients with SZ to ameliorate cognitive function. Recent studies have focused on the use of selective estrogen receptor modulators in the treatment of mental illnesses in the central nervous system (Mirkin and Pickar, 2015). Although these modulators may have a positive influence on cognition and memory and exhibit other effects, the related signaling pathways are not fully understood.

G protein-coupled estrogen receptor 1 (GPER1) is a G protein-coupled receptor that was previously known as GPR30 (Funakoshi et al., 2006). GPER1 is expressed in the plasma membranes of neurons in the hippocampus and prefrontal cortex of the brain (Brailoiu et al., 2007; Akama et al., 2013; Almey et al., 2014). In contrast to estrogen receptor α/β, the classic nuclear function of GPER1 is mainly exerted through activation of nongenomic estrogen signaling pathway members, including the second messengers Ca^2+^ and cyclic adenosine monophosphate, and tyrosine kinase receptor. Studies have shown that estrogen may induce synaptic and cognitive functions through the GPER1-mediated signaling pathway (Gaudet et al., 2015; Waters et al., 2015).

The regulatory effects of GPER1 on the N-methyl-d-aspartate receptor (NMDAR) complex and cognition has received little attention. GPER1 may regulate NMDAR expression and function by affecting NMDAR subunit 2B (NR2B) phosphorylation, which affects brain cognitive function. Potentially, GPER1 can phosphorylate NR2B and regulate the NR2B/CaMKII/CREB pathway. In this study, we aimed to determine whether GPER1 deficiency is related to MK-801-induced SZ dysfunction (such as impaired spatial learning, social communication, and executive function) in mice. The reduced expression of the NR2B subunit in SZ has been investigated in previous work (Kristiansen et al., 2010; Yang et al., 2015; Gulchina et al., 2017; Leung and Ma, 2018). Given that GPER1 is a promising therapeutic target in SZ, the present study investigated changes in the behavioral phenotype and NR2B/CaMKII/CREB signaling pathway related to GPER1 in MK-801-induced SZ.

## Materials and Methods

### Mice

GPER1-KO mice that had been knocked out for 17 nucleotides were obtained from Professor Rong Wei Fang (Shanghai Jiaotong University). Wild-type (WT) mice were provided by the Experimental Animal Center of Ningxia Medical University. Mice (7–8 weeks old; male) were randomly divided into experimental groups and then acclimated for 7 days prior to MK-801 intervention. All mice were housed (four mice/cage) in an animal room at 22 ± 2°C with a 12 h light/dark cycle and lights on at 7:30 AM. They had access to water and food *ad libitum*. Only male WT C57BL/6 and GPER1-KO mice were used in the research. This work was conducted in accordance with the Animal Care Committee of Ningxia Medical University (Protocol approval number: 2019-152). Animal suffering was minimized. WT and KO mice that had been induced with MK-801 (0.6 mg/kg/day, Sigma–Aldrich, Czechia) *via* intraperitoneal injection for 14 consecutive days were designated as the experimental groups: WT SZ and KO SZ (*n* = 14 per group), respectively. WT and KO mice that had been injected with saline (SAL) were designated as controls (WT Con and KO Con, *n* = 14 per group). IntelliCage research was performed with five C57BL/6 and five GPER1-KO MK-801-treated male mice, as well as five C57BL/6 and five GPER1-KO SAL-treated male mice. The classical behavior studies involved nine mice from each group.

### Behavioral Assessment

#### Classical Behavior Studies

**Figure 1:**
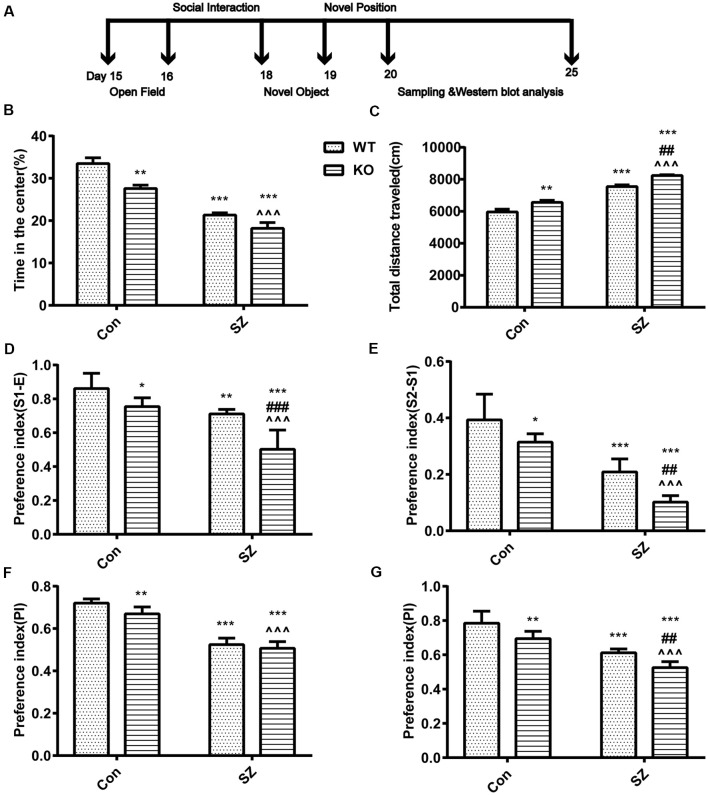
G protein-coupled estrogen receptor 1 (GPER1) deficit increases hyperlocomotion and anxiety-like behavior. **(A)** Classical behavioral experiment design schedule. **(B)** Time spent in the center area among different groups of mice. **(C)** Total distances traveled during the open field test are depicted. **(D,E)** Performance on the social interaction test. Different groups discriminated against the novel individual during the social recognition task. Compared with wild-type (WT) Con mice, knockout (KO) Con mice, and WT schizophrenia (SZ) mice showed less preference for the two unfamiliar mice. Compared with KO Con mice, KO SZ mice showed less preference for the two unfamiliar mice. Compared with WT SZ, KO SZ mice showed less preference for the two unfamiliar mice. **(F)** Novel object preference index (PI). Compared with WT Con mice, KO SZ and KO Con mice spent less time exploring the novel object during the test trial phase. **(G)** Novel placement PI. WT SZ and KO Con mice spent less time exploring novel placement during the test trial phase than WT Con mice. KO SZ mice showed less preference for novel placement than KO Con and WT SZ mice. Data represent the mean ± SD of *n* = 9 mice/group and were analyzed *via* two-way ANOVA followed by *post hoc* test (*p < 0.05, **p < 0.01, ***p < 0.001; ^##^*P* < 0.01, ^###^*p* < 0.001; ^∧∧∧^*p* < 0.001; *WT Con vs. WT SZ, KO Con, KO SZ; ^#^WT SZ vs. KO SZ; ^∧^KO Con vs. KO SZ).

**Figure 2:**
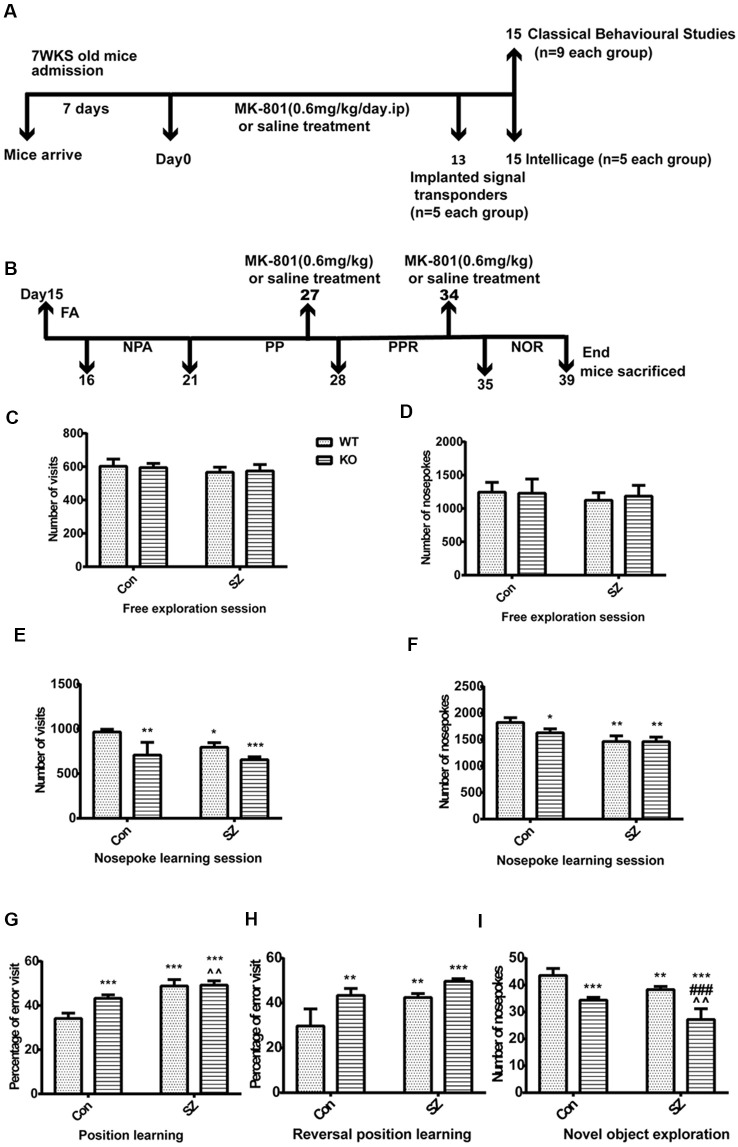
**(A)** Drug administration and the behavioral experiment protocol in this study. **(B)** Intellicage experiment design schedule for assessing multiple cognitive domains with advantages in the validation and characterization of expected cognitive deficits. FA, free adaptation; NPA, nose-poke adaptation; PP, place preference; PPR, place preference reversal; NOR, novel object recognition. **(C,D)** Free exploratory session. No difference was observed among the four groups in terms of the number of visits and number of nose pokes. **(E,F)** Nosepoke learning session, including the number of visits and nose pokes. Compared with those in the WT Con group, the number of visits decreased and the number of nose pokes remained the same in the other groups. **(G)** Position learning. The WT SZ and KO SZ groups showed a higher percentage of erroneous visits than the WT Con group. The KO Con group showed a higher percentage of erroneous visits than the WT Con group. **(H)** Reversal position learning. KO SZ, KO Con, and WT SZ mice showed a higher percentage of erroneous visits than WT Con mice. **(I)** Novel object exploration. WT Con mice had a higher number of nose pokes than the other groups. Data represent the mean ± SD of *n* = 5. Data were analyzed *via* two-way ANOVA followed by Bonferroni *post hoc* test (*p < 0.05, **p < 0.01, ***p < 0.001; ^###^*p* < 0.001; ^∧∧^*p* < 0.01; *WT Con vs. WT SZ, KO Con, KO SZ; ^#^WT SZ vs. KO SZ; ^∧^KO Con vs. KO SZ).

On days of classical behavioral testing, mice were transferred to the behavioral facility 24 h in advance to begin the behavioral assessment for adaptation. All classical behavioral procedures (Figures 1A, 2A) were performed between 06:30 and 11:30 AM (experimental preparation stage: 6:30–7:30 AM and formal experiment stage: 7:30–11:30 AM).

#### Open Field Test

The mice were tested in a Plexiglass open field (OF) after 14 successive days of MK-801 administration to quantify stereotypical behavior and general locomotor activity. Mice were placed in a square OF arena (50 × 50 × 50 cm) and allowed to move freely for 10 min. The time that each mouse spent in the middle of the OF and total distance moved were recorded by using an automatic video tracking system (Smart version 3.0; Panlab, S.L.U., Barcelona, Spain).

#### Social Interaction Test

Mice were tested in a sociability testing apparatus 1 day after the OF test. Social communication and social response to new individuals were evaluated by using a three-chamber apparatus. The three-chamber test consisted of three stages. First, a mouse was transferred to the three-chamber polycarbonate facility and allowed to explore freely for 10 min for habituation. Second, the test mouse was transferred gently to the middle area, and the door on both sides was closed. An unfamiliar, age-matched male mouse was placed in a small cage in the upper area. The doors on both sides of the gates were opened, and the test mouse was allowed to explore for 10 min at will. Last, the test mouse was transferred into the middle area. Both gates were closed again, and a second, unfamiliar age-matched male mouse was placed in the small cage in the lower area. The social capability of mice was expressed by using the preference index (PI).

$${\text{PI}}_{\text{S1-Em}}=(A-B)/(A+B)$$

PI_S1-Em_: Social communication capability; A: Time spent in empty cage; B: Time spent with an unfamiliar, age-matched male mouse.

$${\text{PI}}_{\text{S2-S1}}=(\text{C}-B)/(\text{C}+B)$$

PI_S2-S1_: New social individual’s capability; B: Time spent with an unfamiliar, age-matched male mouse.

C: Time spent with a second, unfamiliar age-matched male mouse.

#### Novel Object Recognition Test

Mice were subjected to the novel object recognition test (NORT) at 1 day after the social interaction test. The NORT was used to test the animal’s ability to recognize a novel object (position) and preference for novel objects (position; Binyamin et al., 2019) in a familiar environment. The preference for a novel object (position) was expressed as PI as follows: (Time_novel object(position)_/(Time_novel object(position)_+ Time_familiar object(position)_; Loh et al., 2015; Dos et al., 2019; Kárpáti et al., 2019; Liu et al., 2019). A mouse that exhibited less than 2 s of the total time exploring objects (position; Time_novel object(position)_+ Time_ familiar object(position)_) was excluded from the analysis. Object recognition testing occurred in two stages, namely, novel object recognition and position recognition. The mice were allowed to explore a field with two identical objects for 5 min during a familiarization trial. This experiment was performed to test the learning and memory of mice for novel objects or positions under free movement. Nonspatial learning and memory capability were evaluated *via* the NORT to verify the effect of GPER1-KO on the recognition and memory functions of SZ mice. After subjecting the mice to 5 min of familiarization and training, the familiar object was replaced with a novel object. The NORT was performed after the mice had rested for 5 min. During the training phase of the novel position recognition test, a mouse was placed in the experimental field. Two identical objects were placed on the same side of the experimental field. During the 5 min training phase, the mouse could identify two objects. One object was placed at the opposite corner of the other object before the test. The novel position recognition test was carried out. The identification time in this experiment was set as 5 min. The PI of the novel object (position) was used to indicate the ability of the mouse to explore the novel object (position).

PI=N/(N+F)

N: Exploring new objects (positions) time; F: Exploring old object (position) time.

### Intellicage

The Intellicage test was performed with mice by using the IntelliCage system, an automated apparatus that monitors the cognitive behavior of multiple mice in a normal living environment (NewBehavior AG^1^). The system contained four intelligent learning condition corners. Each corner had two drinking bottles, and the mouse triggered a sensor chip to open the door by nose-poking and drinking freely. The system automatically recorded the numbers and times the door was opened and the water bottle nozzle was licked. It traced the behavior of the mouse through a transceiver. The IntelliCage system was used to set different experimental procedures to monitor the general activity, spatial learning, and executive function of mice automatically. At 14 days after MK-801 administration, a miniature signal sensor was implanted, and the mice were transferred to an IntelliCage device. During the experiment process, a single administration of MK-801 (0.6 mg/kg) was injected to keep the SZ-like symptoms on days 27 and 34, respectively (Figures 2A,B). The general activity, spatial learning, and executive function of mice were detected. The Intellicage system test protocol included five phases: simple adaptation, nose-poke adaptation, place preference learning, reversal learning, and novel object cognition. (1) Free adaptation stage: all mice could enter the cage and obtain water freely, and all corners and doors were open. Before starting the test programs, the mice were allowed to habituate for 1 day in the IntelliCage with free access to food and water. Following habituation, the mice were trained to obtain access to drinking spouts in all four corners after nose-poking. (2) Nose-poke adaptation stage: for 5 days, all doors were closed, and mice had to open the appropriate door through nose-poking to drink. The door remained open for 7 s. The door opened only when mice visited. Therefore, the mice could gain access to water when visiting. The least-preferred corners by each mouse were identified to guide the design of the next module. (3) Place-learning stage: this phase was similar to phase 2, but the mice could only access water from one corner. Spatial location learning was tested to explore the changes in the spatial positioning and recognition memory of mice. The test time was 7 days. The corners for which the mice showed the least preference for nose-poking were set as “correct,” and the remaining corners were set as “wrong.” Although the experimental mice could poke all corners, the door to the drinking water would open only when the mice poked the “correct” corner. Position-learning capability was evaluated by calculating the error rate of the mice. (4) Reversal learning stage: the identified water corner was replaced by the corner opposite to the one in the place-learning phase. For 7 days, the diagonal corners of the “correct” corners in the spatial position learning of mice were set as the “correct” corners, and the remaining corners were set as the “wrong” corners. The mice may enter all corners at will, and their exploration activities, error rates, and capability to explore new “correct” corners were analyzed. (5) Novel object recognition stage: LED lights in one corner were turned on randomly, and mice could enter and exit all corners and drink water freely. The LED lights in one corner were turned on daily, and the LED lights in different corners were turned in a counter-clockwise direction to monitor the drinking times of mice in the corners where the LED lights were lit. The preference of mice for “new things” were analyzed for 4 days.

### Western Blot Analysis

#### One Week After the Object Recognition Test, Half of the Animals Were Weighed and Decapitated

The mice were sacrificed 1 day after the completion of the classical behavioral tests, and their brains were dissected and isolated. The hippocampus of each mouse was homogenized on ice by using a tissue homogenizer with RIPA buffer. After 15 min of centrifugation at 12,500 rpm and 4°C, the protein concentration was tested *via* the Bradford method. A certain volume of loading buffer was added to each protein sample for denaturation at 100°C for 6 min. After performing SDS–polyacrylamide gel electrophoresis, the targeted proteins were transferred onto PVDF membranes and then blocked with 1% BSA for 1 h. The membranes were incubated with primary antibodies at 4°C overnight (8–12 h), washed with TBST three times, and incubated with secondary antibodies for 1 h at room temperature. Target bands were scanned and analyzed by using the Odyssey Infrared imaging system (Odyssey, LICOR). The following primary antibodies were utilized: mouse anti-NMDAR2B (Abcam, ab181102, 1:500), rabbit anti-phospho-NMDAR2B (Abcam, ab181102, 1:1,000; sites: Tyr1070), mouse anti-CaMKII-α (Cell Signaling Technology, #50049, 1:1,000), rabbit anti-phospho-CaMKII-α (Cell Signaling Technology, #12716, 1:1,000, sites: Thr286), rabbit anti-CREB (Abcam, ab32515, 1:2,000), rabbit anti-phospho-CREB (Abcam, ab32096, 1:1,000, sites: S133), and rabbit anti-β-actin (ZSGB-bio, ZM-0001, 1:1,000).

### Data Analysis

Data were analyzed by using SPSS 24.0 software. Unpaired two-tailed *t*-test and two-way ANOVA with Bonferroni or Tukey–Kramer multiple comparison *post hoc* tests were used to determine differences among groups. *P*-values less than 0.05 were considered statistically significant.

## Results

### Behavioral Studies

#### OF Test

GPER1 knockdown and MK-801 treatment only slightly influenced stereotypical behaviors (*F*_(1,32)_ = 1.563, *p* = 0.22, two-way ANOVA; Figure 1B) and hyperlocomotor activity (*F*_(1,32)_ = 0.175, *p* = 0.68, two-way ANOVA; Figure 1C) in the OF test. Meanwhile, our results demonstrated that GPER1-KO mice had higher total distance (*F*_(1,32)_ = 28.46, *p* < 0.001, two-way ANOVA; Figure 1C) and spent less time in the central area (*F*_(1,32)_ = 17.36, *p* < 0.001, two-way ANOVA; Figure 1B) than WT Con mice. WT SZ mice spent less time in stereotypical behavior (*F*_(1,32)_ = 98.75, *p* < 0.001, two-way ANOVA; Figure 1B) and significantly higher locomotion (*F*_(1,32)_ = 28.46, *p* < 0.001, two-way ANOVA; Figure 1C) than WT Con mice. *Post hoc* analysis showed that GPER1-KO exacerbated the impairment of hyperlocomotion (*t* = 7.873, *p* < 0.001; Figure 1C) and anxiety of mice (*t* = 12.96, *p* < 0.001; Figure 1B) with SZ induced by MK-801. Additionally, KO SZ mice showed increased total distance (*t* = 5.589, *p* < 0.001; *t* = 12.55, *p* < 0.001; Figure 1C) and spent less time in the central area (*t* = 3.677, *p* = 0.002; *t* = 5.956, *p* < 0.001; Figure 1B) than WT SZ and KO Con mice.

#### Social Interaction Test

In the second phase, KO Con (*F*_(1,32)_ = 36.59, *p* < 0.001, two-way ANOVA; Figure 1D) and WT SZ (*F*_(1,32)_ = 59.44, *p* < 0.001, two-way ANOVA; Figure 1D) mice showed less preference for the first unfamiliar mouse than WT Con mice. In the third stage, the KO Con group (*F*_(1,32)_ = 26.62, *p* < 0.001, two-way ANOVA; Figure 1E) and WT SZ (*F*_(1,32)_ = 121.2, *p* < 0.001, two-way ANOVA; Figure 1E) showed less preference for the second unfamiliar mouse than the WT Con group. GPER1 knockdown and MK-801 treatment had no effects on sociability (*F*_(1,32)_ = 3.84, *p* = 0.06, two-way ANOVA; Figure 1D) and social novelty recognition (*F*_(1,32)_ = 0.62, *p* = 0.44, two-way ANOVA; Figure 1E). Compared with mice in the WT SZ and KO Con groups, those in the KO SZ group had less preference for the first (*t* = 5.363, *p* < 0.001; *t* = 6.049, *p* < 0.001; Figure 1D) and second unfamiliar mice (*t* = 6.258, *p* < 0.001; *t* = 17.13, *p* < 0.001; Figure 1E). KO SZ mice exhibited abnormal social interaction ability with their conspecifics and novel conspecifics, indicating that GPER1 KO impaired social interaction and novelty recognition in SZ mice.

#### NORT

The WT SZ group showed a significantly lower preference for novel objects (*F*_(1,32)_ = 343.2, *p* < 0.001, two-way ANOVA; Figure 1F) and placement (*F* = 122.8, *p* < 0.001, two-way ANOVA; Figure 1G) than the WT Con group. Compared with WT Con mice, KO Con mice had less preference for novel objects (*F*_(1,32)_ = 12.24, *p* = 0.001, two-way ANOVA; Figure 1F) and placement (*F* = 32.94, *p* < 0.001, two-way ANOVA; Figure 1G). Meanwhile, the ability of KO SZ mice to identify novel objects was lower than that of KO Con group mice (*t* = 10.82 *p* < 0.001; Figure 1F). Compared with the WT SZ group, the KO SZ group showed reduced capability to recognize novel placement (*t* = 6.265, *p* < 0.001; Figure 1G). There was no interaction of GPER1 knockdown and MK-801 treatment of novel object (*F*_(1,32)_ = 1.563, *p* = 0.22, two-way ANOVA; Figure 1F) and novel position (*F*_(1,32)_ = 0.175, *p* = 0.68, two-way ANOVA; Figure 1G).

#### Intellicage Behavioral Experiment

We used the Intellicage system to further investigate the behavioral differences of mice in different groups. The free adaptation phase showed no difference among the four groups in terms of the number of visits and number of nose pokes. There was no statistically significant difference between-group × within-group interaction (Figures 2C,D) observed. During the nosepoke adaptation phase, the number of visits significantly differed among the four groups and was lower in GPER1 KO mice (*F*_(1,16)_ = 31.62, *p* < 0.001, two-way ANOVA; Figure 2E) and WT SZ group (*F*_(1,16)_ = 9.83, *p* = 0.006, two-way ANOVA; Figure 2E) than in WT Con mice. During this phase, the number of visits in the KO Con group was higher than that in the KO SZ group (*t* = 2.78, *p* = 0.0652; Figure 2E). WT SZ mice exhibited a higher number of visits than KO SZ mice (*t* = 1.021, *p* = 0.8572; Figure 2E). The number of nose pokes showed lower degrees of decline in KO Con (*F*_(1,16)_ = 5.773, *p* = 0.029, two-way ANOVA; Figure 2F) and WT SZ mice (*F*_(1,16)_ = 41.91, *p* < 0.001, two-way ANOVA; Figure 2F) compared with those in WT Con mice. At the same time, the number of nose pokes in the KO SZ group significantly decreased (*t* = 2.957, *p* = 0.048; Figure 2F) compared with that in the KO Con group. GPER1 knockdown and MK-801 treatment significantly affected the number of nose pokes (*F*_(1,16)_ = 5.446, *p* = 0.033, two-way ANOVA; Figure 2F). The place learning results showed that the percentage of erroneous visits was higher in the WT SZ group (*F*_(1,16)_ = 102.7, *p* < 0.0001, two-way ANOVA; Figure 2G) than in the WT Con group, and KO Con mice exhibited a higher rate of errors in this stage than WT Con mice (*F*_(1,16)_ = 21.84, *p* = 0.0003, two-way ANOVA; Figure 2G). A significant effect was seen in the following parameters: genotype × MK-801 interaction in position learning (*F*_(1,16)_ = 18.31, *p* = 0.0006, two-way ANOVA; Figure 2G). Meanwhile, KO SZ mice had a higher percentage of erroneous visits than KO Con mice (*t* = 4.14, *p* = 0.0038; Figure 2G). In the reversal learning phase, the percentage of erroneous visits in the KO SZ group was higher than that in the WT SZ group (*t* = 7.71, *p* < 0.001; Figure 2H). WT Con mice showed a lower percentage of erroneous visits than KO Con mice (*F*_(1,16)_ = 30.66, *p* < 0.0001, two-way ANOVA; Figure 2H) and WT SZ mice (*F*_(1,16)_ = 25.17, *p* = 0.0001, two-way ANOVA; Figure 2H). There was no interaction of GPER1 knockdown and MK-801 treatment of the reversal learning phase (*F*_(1,16)_ = 2.835, *p* = 0.11, two-way ANOVA; Figure 2H). The results from the new object recognition stage showed that the number of nose pokes in the WT SZ (*F*_(1,16)_ = 30.91, *p* < 0.0001, two-way ANOVA; Figure 2I) and KO Con groups (*F*_(1,16)_ = 81.44, *p* < 0.0001, two-way ANOVA; Figure 2I) was lower than that in the WT Con group. The KO SZ group displayed a lower number of nose pokes than the WT SZ group (*t* = 5.951, *p* < 0.001; Figure 2I). Additionally, our results revealed the weak effect of GPER1 knockdown and MK-801 treatment (*F*_(1,16)_ = 0.7807, *p* = 0.39, two-way ANOVA; Figure 2I).

### Western Blot Analysis

**Figure 3:**
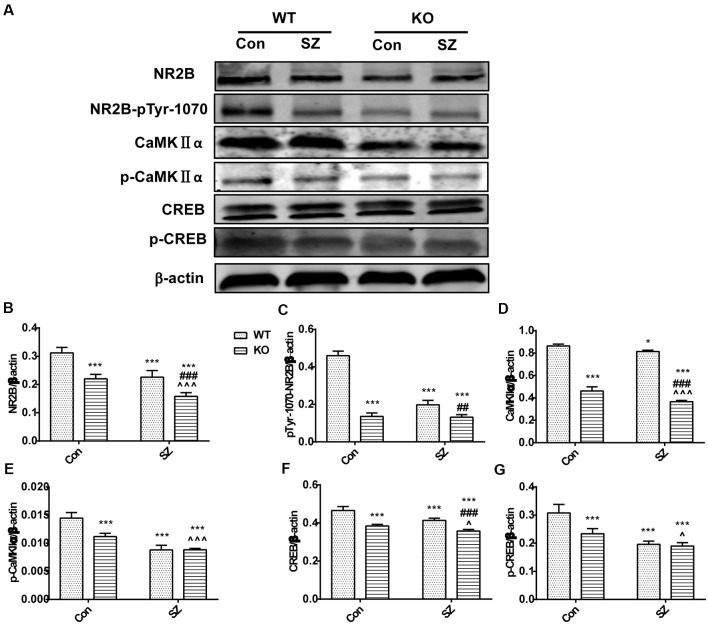
GPER1 deficiency downregulated the NMDAR subunit 2B (NR2B)/CaMKII/CREB signaling cascade in the hippocampus of the mouse models of SZ-like phenotypes. **(A)** Representative immunoblots showing the protein levels of NR2B, pTyr-1070-NR2B, p-CaMKIIα, CaMKIIα, CREB, and phospho-CREB in the hippocampus detected by Western blot analysis. **(B–G)** Histograms showing the quantification of protein band densities that had been normalized to β-actin. Data are presented as mean ± SD of *n* = 5 mice/group. Data were analyzed *via* two-way ANOVA followed by Bonferroni *post hoc* test (*p < 0.05, ***p < 0.001; ^##^*p* < 0.01, ^###^*p* < 0.001; ^∧^*p* < 0.05, ^∧∧∧^*p* < 0.001; *WT Con vs. WT SZ, KO Con, KO SZ; ^#^WT SZ vs. KO SZ; ^∧^KO Con vs. KO SZ).

We tested several key proteins of the NR2B/CaMKII/CREB pathway in the hippocampus of GPER1-KO mice to investigate the possible mechanism by which MK-801 induced SZ-like symptoms (Figure 3A). GPER1 knockdown and MK-801 treatment significantly influenced the expression levels of the following proteins: CREB (*F*_(1,16)_ = 4.761, *p* = 0.0444, two-way ANOVA; Figure 3F), p-CREB (*F*_(1,16)_ = 15.11, *p* = 0.0013, two-way ANOVA; Figure 3G), CaMKIIα (*F*_(1,16)_ = 5.539, *p* = 0.0317, two-way ANOVA; Figure 3D), p-CaMKIIα (*F*_(1,16)_ = 27.08, *p* < 0.0001, two-way ANOVA; Figure 3E), and NR2B-pTyr-1070 (*F*_(1,16)_ = 199.3, *p* < 0.0001, two-way ANOVA; Figure 3C). These results suggested that GPER1 deficiency might cause a reduction in the levels of proteins related to the NR2B/CaMKII/CREB signaling pathway in the mouse hippocampus, and MK-801 aggravated this change. Meanwhile, we found there was no interaction of GPER1 knockdown and MK-801 treatment of NR2B expression (*F*_(1,16)_ = 2.182, *p* = 0.1591, two-way ANOVA; Figure 3B). Overall, these results proved that the expression levels of NR2B, NR2B-pTyr-1070, CaMKIIa, p-CaMKIIa, CREB, and p-CREB decreased in the presence of SZ symptoms (Figures 3B–G). Therefore, the NR2B/CaMKII/CREB signaling pathway in the hippocampus might be involved in the behavioral effects induced by MK-801 in KO SZ mice.

## Discussion

SZ is a neuropsychiatric disease with gender differences (Bratek et al., 2016). Female patients with SZ are usually older and have milder symptoms than male patients with SZ. Clinically, estrogen may improve the cognitive impairment caused by SZ. GPER1, a newly identified estrogen receptor, may regulate the rapid nongenetic effect of estrogen. Studies have proven that GPER1 can promote spatial working memory in ovariectomized animals *via* the systemic injection of a GPER1 agonist (Gurvich et al., 2019). GPER1 can regulate anxiety-related emotions, spatial learning, and memory (Hammond et al., 2009; Kastenberger and Schwarzer, 2014; Kim et al., 2016). NMDAR plays a key role in the etiology and drug treatment of SZ (Lee and Zhou, 2019). Numerous studies have confirmed that NMDAR antagonists induce SZ-related behavior (Jeon et al., 2017). Meanwhile, our previous studies (Ding et al., 2017, 2019) and other work (Seillier and Giuffrida, 2009; Jacklin et al., 2012; Karamihalev et al., 2014; Li et al., 2016) found cognition impairment and alterations in anatomical and neurochemical aspects in a mouse subchronic model of SZ induced by the intraperitoneal injection of MK-801 for 14 consecutive days. Given that SZ is a chronic psychiatric disorder, subchronic administration with MK-801 might better represent the enduring cognition deficits of SZ (Karamihalev et al., 2014; Xiu et al., 2014), meaning it may serve as a robust model for the disease. Thus, the subchronic model of SZ was utilized in this study. Moreover, the dose of MK-801 (0.6 mg/kg/day) was utilized in our previous research (Huang et al., 2018; Ding et al., 2019). Based on the hypothesis of this past study, we evaluated the effects of GPER1 deficiency on the hyperlocomotion, stereotypical behaviors, novel object recognition, social interaction, general activity, spatial learning, and the executive capability of the mouse subchronic model of SZ. We also investigated whether GPER1 deficiency is associated with the expression levels of several proteins in the NR2B/CaMKII/CREB signaling pathway in SZ-like mice induced by MK-801. Previous studies found that the male hippocampus expresses all three major estrogen receptors (ER, ER, and GPER1; Hazell et al., 2009; Loh et al., 2015). Meanwhile, no apparent gender differences were observed in the distribution of GPER1/GPR30 in the adult mouse brain (Hazell et al., 2009). GPER1 is an extranuclear estrogen receptor whose functions in the central nervous system are largely unknown. However, it can activate kinase cascades and accelerate calcium flux within cells (Hadjimarkou and Vasudevan, 2018). Studies have demonstrated that GPER1 signaling in the brain is a response to estrogen, and it performs neuroprotection, cognition, and memory enhancement functions (Vajaria and Vasudevan, 2018). Other studies have reported that GPER1 improves spatial memory and promotes the generation of newborn neurons in the hippocampus; moreover, it may be involved in the release of neurotransmitters (Almey et al., 2015; Briz et al., 2015). Recently, the role of GPER1 in the regulation of mood disorders has been described (Kastenberger and Schwarzer, 2014). Studies on rodents have reported that there is an anti-depressant-like effect of estradiol that is mediated by GPER1, leading to the hypothesis that GPER1 might be an innovative target for the treatment of depression. The serum of patients with generalized anxiety disorder shows increased GPER1 levels, which correlate with the degree of anxiety (Orhan et al., 2018). However, there is no evidence that GPER1 deficiency might aggregate SZ-like behaviors induced by MK-801 *in vivo*. In this study, we performed a classical and intelligent behavioral analysis of mice that received GPER1 knockdown, and our results demonstrated increased hyperlocomotion and stereotypical behaviors, decreased social interaction, impaired novelty exploration, and recognition function, and impaired executive function in GPER1-KO SZ mice compared with their control counterparts. Impairments in learning and memory (Goldman-Rakic, 1994; Stefani and Moghaddam, 2005), hyperlocomotion, and stereotypical behaviors (Morrens et al., 2006; Xiao et al., 2019), and social withdrawal (Kay et al., 1987) are typical SZ symptoms. Our comprehensive behavioral results from learning and memory, social interaction, and executive function tests supported the notion that the administration of MK-801 interfered easily in GPER1-KO mice. Thus, our results suggested that GPER1 knockout aggravated the onset of SZ-like symptoms. We also found that CPER1 deficiency significantly reduced the learning capability and memory of SZ mice. Notably, this study is the first to demonstrate that GPER1 deletion led to further declines in the learning and memory of mice with MK-801-induced SZ. These results implied that the lack of GPER1 aggravated the cognitive impairment of SZ. Our results were consistent with previous studies on molecular changes in patients with SZ and animal models of the hippocampus (Silvestre de Ferron et al., 2015; Yang et al., 2015; Gulchina et al., 2017; Zhou et al., 2018). Notably, MK-801 significantly reduced the expression of NR2B in the mouse hippocampus. Meanwhile, GPER1 deficiency exacerbated the reduction in NR2B protein levels of SZ mice. We found that the expression of NR2B-pTyr-1070 protein was reduced in SZ mice. In GPER1-KO SZ mice, we found a significant decrease in the NR2B-pTyr-1070 protein expression level. This result indicated that GPER1 was related to the regulation of NR2B phosphorylation possibly in SZ mice. However, the mechanisms by which GPER1 regulates NR2B phosphorylation in the hippocampus of mice with SZ requires further investigation.

As a downstream signal factor of the NR2B subunit, CaMKII participates in a variety of signaling cascade responses and is an important mediation center for learning and memory. CaMKIIα is an extremely abundant protein kinase in brain tissue that participates in multiple signaling cascade reactions to regulate learning and memory. Consistent with previous studies, in this work, we found that the protein expression levels of CaMKIIα, phosphorylated CaMKIIα, CREB (Ren et al., 2014), and phosphorylated CREB decreased in SZ mice. We also saw that the protein expression levels of CaMKIIα, phosphorylated CaMKIIα, CREB, and phosphorylated CREB significantly decreased in GPER1-deficient SZ mice. GPER1 deficiency promoted MK-801-induced SZ in mice, indicating that GPER1 deficiency resulted in NR2B and NR2B-pTyr-1070 hypofunction and NR2B/CaMKII/CREB signaling pathway downregulation in SZ mice. The mechanism underlying the further decline of some cognitive abilities in SZ mice caused by GPER1 deletion might partially occur through the downregulation of the NR2B/CaMKII/CREB signaling pathway. This study is the first to evaluate the relationship between GPER1 and the NR2B/CaMKII/CREB signaling pathway in an animal model of SZ. Thus, GPER1 might phosphorylate NR2B and regulate the NR2B/CaMKII/CREB pathway.

## Conclusion

Collectively, the results of this study suggest that GPER1 deficiency can attenuate cognitive function in SZ mice. GPER1 deficiency might promote the degradation of the NR2B/CaMKII/CREB signaling pathway by inhibiting NR2B phosphorylation. Therefore, GPER1 activity may be a new target in the treatment of SZ and other symptoms, such as learning and memory impairment. Future studies should focus on the effect of GPER1 on the pathogenesis of SZ *in vivo* and *in vitro*.

## Data Availability Statement

The raw data supporting the conclusions of this article will be made available by the authors, without undue reservation.

## Ethics Statement

The animal study was reviewed and approved by Ningxia Medical University Animal Research Ethics Board.

## Author Contributions

CZ, QL, JL, and TS conceived and designed the experiments. FW and K-SS conducted the experiments and performed the statistic alanalyses. YS and C-YY provided the experimental animals and assisted with the animal breeding. CZ wrote the manuscript. All authors contributed to the article and approved the submitted version.

## Conflict of Interest

The authors declare that the research was conducted in the absence of any commercial or financial relationships that could be construed as a potential conflict of interest.

## Footnotes

^1^http://www.newbehavior.com
